# On-top-plasty reconstruction for thumb triplication^☆^

**DOI:** 10.1016/j.jpra.2023.08.007

**Published:** 2023-08-17

**Authors:** Kosuke Kuwahara, Sohachi Toriyabe, Masahiro Nakagawa, Hideaki Kamochi

**Affiliations:** a Department of Plastic Surgery, Shizuoka Children's Hospital, Shizuoka, Japan; b Department of Plastic and Reconstructive Surgery, Hamamatsu University School of Medicine, Hamamatsu, Japan; c Department of Plastic and Hand Surgery, Sendai Medical Center, Sendai, Japan

**Keywords:** Radial polydactyly, Triplication, Triplicated thumb, On-top-plasty, Thumb reconstruction

## Abstract

Thumb triplication is a very rare pattern of radial polydactyly that presents as a more complicated form of thumb. Because of its morphological complexity, treatment often requires surgical ingenuity in addition to the conventional surgical treatment algorithms for duplication. We report the case of thumb triplication on the right hand of a 17-month-old boy. We performed on-top-plasty of the ulnar thumb over the intermediate thumb, and achieved a functional and aesthetic thumb. The technique of on-top-plasty is effective for finger reconstruction that maximizes the use of limited tissue and is widely used for treating hand trauma and congenital hand anomalies. In the present case, on-top-plasty contributed most effectively to creating a sufficiently sized thumb, achieving interphalangeal optimization by joining the extra middle phalanx with the proximal phalanx, and securing the first web space by moving the ulnar thumb laterally.

## Introduction

Radial polydactyly is the most common congenital hand anomaly. Most anomalies consist of thumb duplication, and triplication is a very rare pattern that presents a more complicated form of thumb.^2^^,^^3^ Because of its morphological complexity, it is unclassifiable by Wassel's classification system^1^ and surgical ingenuity is often required in addition to the conventional surgical treatment algorithms for duplication.

To establish the most functional and aesthetic outcome, simple resection of the two accessory digits should be avoided; rather, combination of the tissues of each thumb should be considered, to augment the reconstructed thumb.

We performed on-top-plasty of the ulnar thumb over the intermediate thumb after resecting the radial thumb and the hypoplastic distal segment of the intermediate thumb. This method resulted in a thick and long reconstructed thumb with a wide and deep first web and good aesthetic appearance of the hand.

## Case presentation

**Figure 1:**
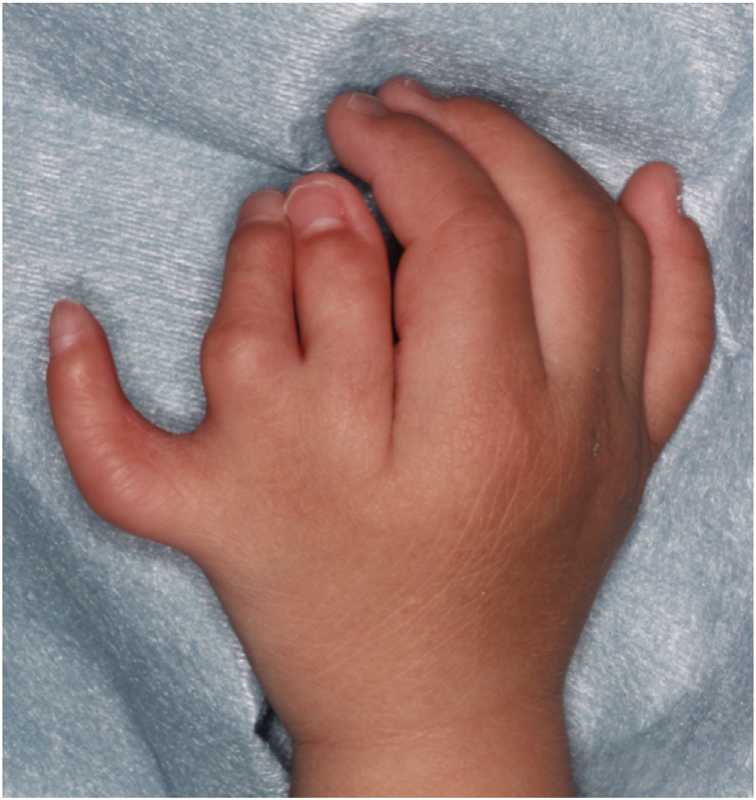
Preoperative image of the right hand.

**Figure 2:**
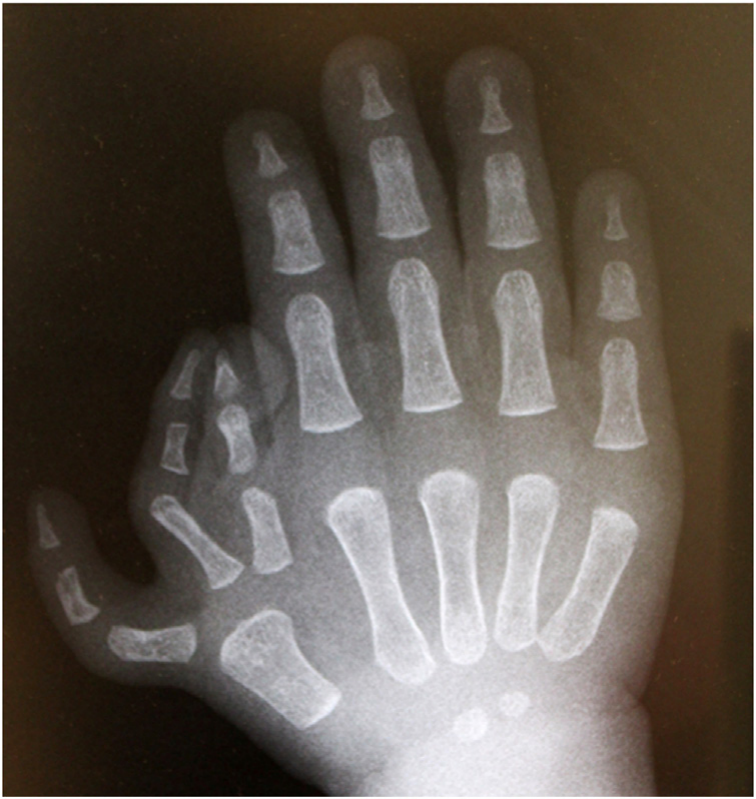
Preoperative radiograph showing triplication, consisting of three triphalangeal thumbs.

We report the case of thumb triplication on the right hand of a 17-month-old boy (Figure 1). The radial thumb and intermediate thumb were distally hypoplastic and deviated. The ulnar thumb was the thickest and longest, and the most cosmetically acceptable, but was located close to the index finger in the first web space. The proximal region had features of simple syndactyly. Radiography confirmed complete triplication consisting of three triphalangeal thumbs that shared a common metacarpal (Figure 2).

**Figure 3:**
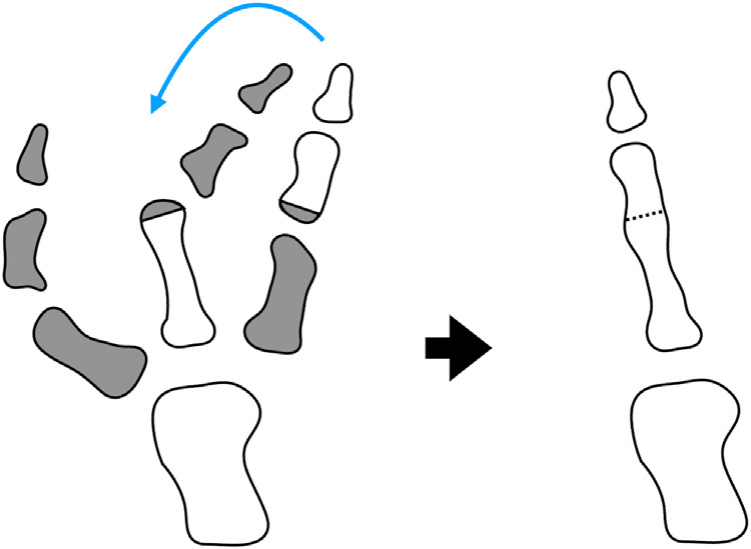
Schema of on-top-plasty based on the preoperative radiograph.

Simple resection of the hypoplastic radial thumb and intermediate thumb would not result in a satisfactory reconstructed thumb. To achieve a functional and aesthetic thumb in addition to a wide and deep first web space, we elected to perform on-top-plasty of the ulnar thumb over the intermediate thumb (Figure 3). First, the radial thumb was resected at the level of the metacarpophalangeal (MP) joint. The abductor pollicis brevis (APB) muscle and extensor pollicis longus (EPL) tendon were identified and isolated to enable later reattachment to the reconstructed thumb. Flexor pollicis longus (FPL) tendon was absent in the radial thumb. Second, dorsal veins, volar neurovascular bundles, and the EPL and FPL tendons were identified for the ulnar thumb, and the ulnar proximal phalanx was enucleated. A pedicled “floating” thumb that allowed lateral movement was then prepared. Finally, the hypoplastic distal segment of the intermediate thumb was resected at the level of the distal metaphysis of its proximal phalanx, and on-top-plasty of the ulnar thumb over the intermediate thumb was performed. EPL and FPL tendons of the intermediate thumb were identified, but were not available due to hypoplasia. APB muscle and the radial EPL tendon were transferred to the reconstructed thumb to enable abduction, alignment correction, and ensure joint stability of this thumb. A wide and deep first web was created by direct skin suturing of the space where the ulnar thumb previously existed. The osteotomy site was pinned with 0.7 mm Kirschner wire for 6 weeks.

**Figure 4:**
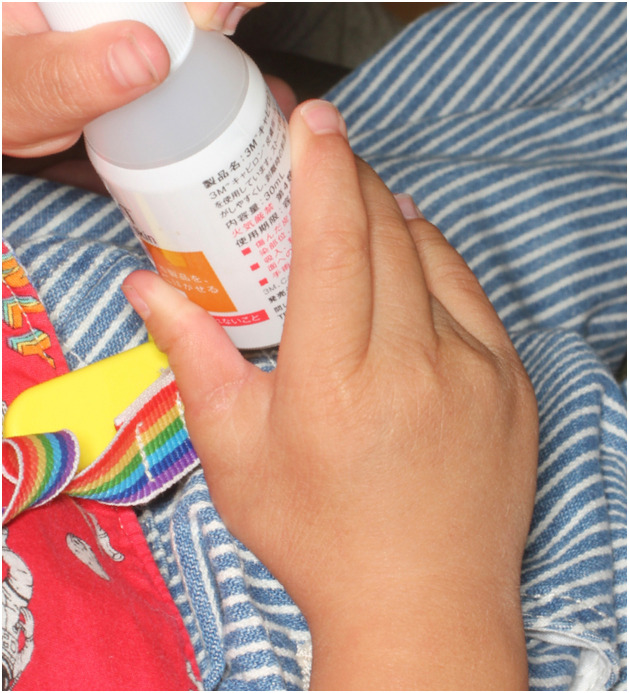
One-year postoperative image shows no problems with grasping or pinching.

At the one-year follow-up after surgery, the thumb retained excellent aesthetic appearance. Despite having a slight radial bend, the oppositional function of the thumb and joint stability were improved and there were no problems with grasping or pinching (Figure 4).

## Discussion

The scarcity of published case reports of thumb triplication indicates that this is a very rare form of radial polydactyly.^2^, ^3^, ^4^, ^5^ Differences in incidence rates between races as well as genetic factors associated with chromosome 7q36.3 have been reported,^5^^,^^6^ but the actual frequency is still unknown. In addition, very few reports have referred to surgical techniques for reconstruction of triplicated thumb.

Due to the very complicated phenotype of triplicated thumb, simple classification by methods such as Wassel's classification system is not suitable. To ensure morphological understanding and treatment planning for the phenotype, the Rotterdam classification reported by Zuidam et al. may be useful.^6^ This classification can simply and accurately describe radial polydactyly, including complex components such as triphalangism and triplication.

As triplication has been reported to be often associated with triphalangism^2^, ^3^, ^4^, ^5^ and there is great variety in the soft tissue conditions, surgical ingenuity is required in addition to conventional surgical treatment algorithms for duplication. However, the treatment goals for thumb reconstruction are identical to those for duplication. Surgeons should always aim to reconstruct an appropriate thumb thickness and length, longitudinal alignment, phalanx number, tendon balance, nail shape, joint stability, and deep and wide first web to establish a functional and aesthetic thumb. In particular, the treatment concept of “spare part surgery” is very important. Simple resection of the two accessory digits should be avoided, and active consideration should be given to combining the tissues of each thumb to augment the reconstructed thumb, using an on-top-plasty or side-to-side plasty such as the Bilhaut–Cloquet procedure.^7^

The on-top-plasty performed in the present patient is an excellent technique for finger reconstruction that maximizes the use of limited tissue and is widely used for treatment of hand trauma and congenital hand anomalies.^8^^,^^9^ In our case, this technique contributed most effectively to creating a sufficiently sized thumb, interphalangeal optimization by joining the extra middle phalanx with the proximal phalanx, and securing the first web space by moving the ulnar thumb laterally. In contrast, Yoneda et al. voiced concerns about the instability of the reconstructed thumb joint after on-top-plasty, suggesting that additional surgery such as arthrodesis may be required.^10^ In the present case, slight radial bending with deformed proximal phalanges including the osteotomy site, and irregular metacarpal bones were observed. Long-term follow-up is necessary to evaluate the possible exacerbation of these deformities.

## Informed consent

Informed consent to include case details and images in our publication was obtained from parents of the patient.

## Ethical statement

Not applicable.

## Conflicts of interest

The authors declare that they have no known competing financial interests or personal relationships that could have appeared to influence the work reported in this paper.
